# Tracing cell fates in embryos

**DOI:** 10.7554/eLife.108962

**Published:** 2025-10-01

**Authors:** Ying Zhang, Qi Chen

**Affiliations:** 1 The Key Laboratory of Cell Proliferation and Regulation Biology, Ministry of Education, College of Life Sciences, Beijing Normal University Beijing China; 2 Molecular Medicine Program, Department of Human Genetics, Division of Urology, Department of Surgery, University of Utah School of Medicine Salt Lake City United States

**Keywords:** polarity, preimplantation development, mouse embryo development, cell fate, Mouse

## Abstract

Differences in the activity of an enzyme called CARM1 influence the timing of blastomere polarization and whether they become part of the embryo or the placenta.

**Related research article** Lamba A, Zhu M, Meglicki M, Czukiewska S, Balasubramaniam L, Hadas R, Weishaupt N, Patel EM, Kavanagh YH, Wang R, Jing N, Zernicka-Goetz M. 2025. Asynchronous mouse embryo polarization leads to heterogeneity in cell fate specification. *eLife* **13**:RP101140. doi: 10.7554/eLife.101140.

Prenatal development is a complex and highly coordinated process that begins at conception. Within the first 24–36 hours, a fertilized human egg starts to divide into two cells, then four, eight, sixteen, and so on. These early cells – which are known as blastomeres – are often assumed to be identical. By the fifth or sixth day, however, the embryo has formed a rapidly dividing, differentiated structure known as a blastocyst.

At this stage, it is thought that cells begin to specialize into the inner cell mass, which will develop into the embryo, and the trophectoderm, the outer layer that gives rise to the placenta and supporting tissues (Chen et al., 2018). But how does a single fertilized mammalian egg transition into a structured embryo with distinct lineages, without relying on the well-defined chemical gradients that guide development in frogs or worms?

For decades, the prevailing view from mouse studies was that cell fate is not determined until late in the eight-cell stage, when each cell establishes a ‘cap’ of polarity proteins that defines its orientation. When the embryo next divides, the cells that retain this cap remain on the surface of the embryo and go on to form the future trophectoderm, whereas the cells without this cap become the inner cell mass. This model assumes that all cells are identical up until polarity emerges, and that symmetry is broken after the eight-cell stage. But are earlier cells truly identical?

Researchers have found that, at the four-cell stage, mouse blastomeres already show differences in the levels and activity of an enzyme called CARM1, which influences gene expression by altering how DNA is packaged into chromatin. Cells with lower CARM1 activity tend to become the trophectoderm, while those with higher levels tend to form the inner cell mass (Torres-Padilla et al., 2007). Yet it remained unclear whether these early molecular asymmetries persist into the eight-cell stage, and whether they play a role in cell fate determination.

Now, in eLife, Magdalena Zernicka-Goetz (Cambridge University and Caltech) and colleagues – including Adiyant Lamba and Meng Zhu as joint first authors – report new insights into this longstanding question (Lamba et al., 2025). Through high-resolution live imaging and cell lineage tracing of mouse embryo cells, the researchers – who are based at Cambridge, Caltech, Harvard Medical School and the State Key Laboratory of Cell Biology in Shanghai – challenge the long-standing view that polarity arises all at once. Instead, they show that polarization occurs asynchronously: some cells polarize early, others later, and this difference in timing influences fate: early polarizers tend to form the trophectoderm, while later ones are more likely to contribute to the inner cell mass. Crucially, they show that this asynchrony is linked to differences in CARM1 activity at the four-cell stage. Cells with lower CARM1 levels are predisposed to polarize early, providing the first mechanistic link between early molecular differences in the four-cell stage and the timing of polarity in the eight-cell stage (Figure 1).

**Figure 1.**
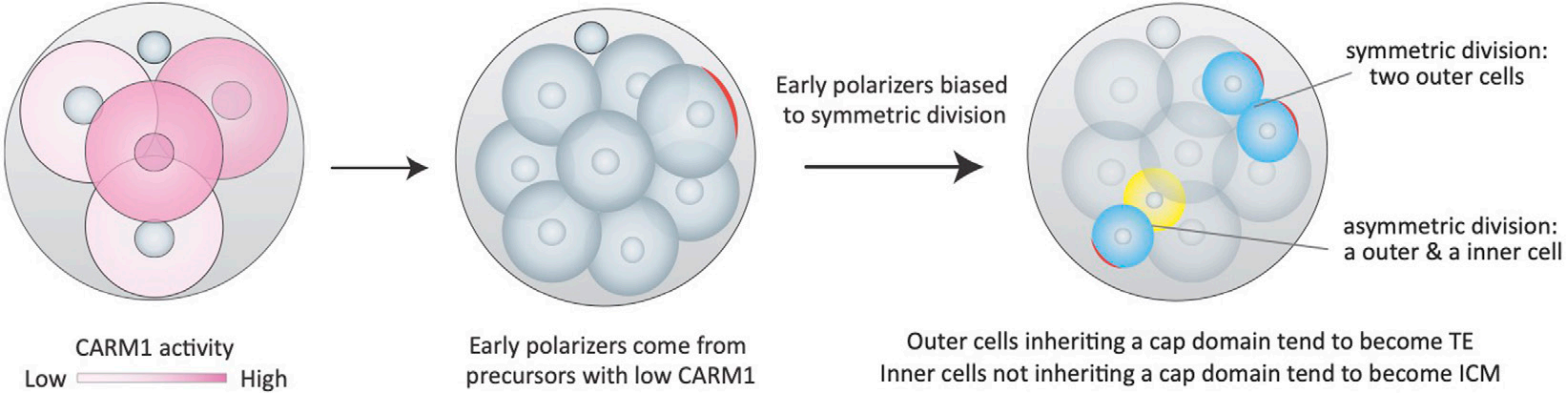
Schematic illustration showing how differences in CARM1 activity can lead to different timings of blastomere polarization and cell fates. Left: During the four-cell stage, some blastomeres have low levels of CARM1 activity (light pink), and some have high levels (dark pink). Small grey circles represent the polar bodies, which are generally seen inside the zona pellucida. Middle: The cells with lower levels of CARM1 activity tend to become early polarizers during the eight-cell stage. These cells are broader in shape, with the nucleus situated near the cell surface (as can be seen in the cell with the red cap). Right: Early polarizers also have more symmetric cell divisions (71.7% symmetric; 28.3% asymmetric; two blue cells at top right), with about 80% of the descendant cells from early polarizers becoming part of the future trophectoderm (TE). Cells that polarize later favor asymmetric cell division (38.7% symmetric, 61.3% asymmetric; blue and yellow cell, bottom left), with about two-thirds of descendant new cells from late polarizers becoming part of the future trophectoderm, and about one-third becoming part of the inner cell mass (ICM).

Furthermore, the early-polarizing cells display different appearances and behaviors. They are broader in shape, with nuclei situated near the surface. They start expressing a protein essential for the formation of the trophectoderm (Cdx2) earlier, and when they divide, they are more likely to produce two outer daughter cells. Consequently, over 80% of their progeny contribute to the trophectoderm, compared to about 68% of late polarizers (Figure 1).

This bias results from protein networks that stabilize the cap through structural filaments called keratins. Importantly, reduced CARM1 activity at the four-cell stage encourages this outcome by increasing levels of BAF155, a chromatin regulator that boosts keratin expression. In this way, subtle molecular asymmetries at the four-cell stage set the foundation for uneven polarization at the eight-cell stage, laying the groundwork for later lineage differences.

An obvious question is: what triggers the initial differences in CARM1 at the four-cell stage? One clue is to be found in a regulatory RNA called *LincGET*, which has been unevenly distributed since the two-cell stage. This RNA promotes nuclear activity in CARM1 and biases gene expression towards the inner cell mass (Wang et al., 2018). More broadly, single-cell analyses indicate that small transcriptional variations emerge as early as the two-cell stage, possibly due to imperfect cleavage, uneven inheritance of RNA or proteins, or contributions from sperm (such as RNAs and mitochondrial proteins; Shi et al., 2015; Chen et al., 2018; Chen et al., 2016; Luo et al., 2013). Such early molecular heterogeneity, though subtle, may then be amplified by zygotic genome activation and regulatory feedback, helping explain how initial stochastic events are inevitably reinforced to bias cell fate during development (Goolam et al., 2016; Shi et al., 2015; White et al., 2016).

Beyond gene regulation, physical forces may also contribute to heterogeneity. The embryo’s shell, the zona pellucida, is often oval rather than spherical (Kurotaki et al., 2007). In a four-cell embryo, clustering into a tetrahedral arrangement can result in uneven mechanical compression on individual cells, depending on their position along the longer axis of the zona pellucida. These geometrical and mechanical differences may, in turn, influence nuclear positioning, chromatin organization and regulators such as CARM1. Clarifying how biomechanical effects intersect with early molecular asymmetries in the mammalian embryo will therefore be an important direction for future research.

Taken together, the findings of Lamba et al. reinforce the view that symmetry breaking in the mammalian embryo is not a sudden event. Instead, subtle heterogeneities established during early divisions are gradually amplified to guide cell fate, while maintaining plasticity. By showing that polarization occurs asynchronously, the researchers link early molecular heterogeneity to later fate allocation. Extending these insights to human embryos could improve our understanding of embryo quality and its relationship to successful pregnancy outcomes. Moreover, variability in polarization timing may hold new predictive value for developmental potential.
